# The Facilitative Effect of Transcranial Direct Current Stimulation on Visuospatial Working Memory in Patients with Diabetic Polyneuropathy: A Pre–post Sham-Controlled Study

**DOI:** 10.3389/fnhum.2016.00479

**Published:** 2016-09-28

**Authors:** Yi-Jen Wu, Philip Tseng, Han-Wei Huang, Jon-Fan Hu, Chi-Hung Juan, Kuei-Sen Hsu, Chou-Ching Lin

**Affiliations:** ^1^Institute of Clinical Medicine, College of Medicine, National Cheng Kung UniversityTainan, Taiwan; ^2^Department of Neurology, National Cheng Kung University Hospital, College of Medicine, National Cheng Kung UniversityTainan, Taiwan; ^3^Graduate Institute of Humanities in Medicine, Taipei Medical UniversityTaipei, Taiwan; ^4^Brain and Consciousness Research Center, Shuang Ho Hospital, Taipei Medical UniversityNew Taipei City, Taiwan; ^5^Department of Psychology and Institute of Cognitive Science, College of Social Sciences, National Cheng Kung UniversityTainan, Taiwan; ^6^Institute of Cognitive Neuroscience, National Central UniversityJhongli, Taiwan; ^7^Department of Pharmacology, College of Medicine, National Cheng Kung UniversityTainan, Taiwan

**Keywords:** diabetic polyneuropathy, visuospatial working memory, transcranial direct current stimulation, dorsolateral prefrontal cortex, nerve conduction velocity, Corsi block tapping task, mild cognitive impairment

## Abstract

Diabetes mellitus can lead to diabetic polyneuropathy (DPN) and cognitive deficits that manifest as peripheral and central neuropathy, respectively. In this study we investigated the relationship between visuospatial working memory (VSWM) capacity and DPN severity, and attempted to improve VSWM in DPN patients via the use of transcranial direct current stimulation (tDCS). Sixteen DPN patients and 16 age- and education-matched healthy control subjects received Wechsler Adult Intelligence Scale-Fourth Edition (WAIS-IV) and Montreal Cognitive Assessment (MOCA) for baseline cognitive assessment. A forward- and backward-recall computerized Corsi block tapping task (CBT), both with and without a concurrent motor interference task was used to measure VSWM capacity. Each DPN patient underwent a pre-treatment CBT, followed by tDCS or sham treatment, then a post-treatment CBT on two separate days. We found that although patients with severe DPN (Dyck’s grade 2a or 2b) showed comparable general intelligence scores on WAIS-IV as their age- and education-matched healthy counterparts, they nonetheless showed mild cognitive impairment (MCI) on MOCA and working memory deficit on digit-span test of WAIS-IV. Furthermore, patients’ peripheral nerve conduction velocity (NCV) was positively correlated with their VSWM span in the most difficult CBT condition that involved backward-recall with motor interference such that patients with worse NCV also had lower VSWM span. Most importantly, anodal tDCS over the right DLPFC was able to improve low-performing patients’ VSWM span to be on par with the high-performers, thereby eliminating the correlation between NCV and VSWM. In summary, these findings suggest that (1) MCI and severe peripheral neuropathy can coexist with unequal severity in diabetic patients, (2) the positive correlation of VSWM and NCV suggests a link between peripheral and central neuropathies, and (3) anodal tDCS over the right DLPFC can improve DPN patients’ VSWM, particularly for the low-performing patients.

## Introduction

Diabetes mellitus (DM) causes multiple complications, and among which diabetic polyneuropathy (DPN) occurs in up to 50% of the patients with long duration of DM (Tesfaye et al., 2011). Patients with DPN experience numbness, paresthesia, allodynia, and weakness over distal limbs (Callaghan et al., 2012). Critically, cognitive deficits in patients with DM can emerge as an important issue (Exalto et al., 2012; Pearce et al., 2012) as recent studies have revealed that DM can be a significant risk factor for dementia, including both vascular dementia and Alzheimer’s disease (Umegaki, 2014). These cognitive impairments include mental and motor slowing, and worsened executive functioning such as planning, problem-solving and working memory (WM). Patients with type 2 DM can show learning and WM decrements (McCrimmon et al., 2012), which are associated with prefrontal lobe dysfunction and reduced glucose metabolism, as well as decreased gray matter density and white matter integrity in type 2 DM (Garcia-Casares et al., 2014).

Although, the abovementioned cognitive deficits in DM patients are usually mild and rarely meet the clinical criteria of dementia, they widely affect the patients’ working performances and daily activities (McCrimmon et al., 2012). This is especially true in the context of visuospatial working memory (VSWM), a short-term memory buffer that stores object locations so that we remember what we see and can plan goal-directed actions despite of continuous visual disruptions such as blinks and eye movements (McAfoose and Baune, 2009; Tseng and Bridgeman, 2011). Consequently, VSWM is critical to many daily tasks and has been demonstrated as a good predictor of fluid intelligence (Fukuda et al., 2010), wayfinding (Nori et al., 2009), and driving safety (Anstey et al., 2012), all of which can severely impact patients’ quality of life if impaired. Previous functional neuroimaging studies have pointed to the activation of the dorsal stream, including dorsolateral prefrontal cortex (DLPFC), posterior parietal cortex (PPC), and frontal eye field (FEF) to be highly relevant to VSWM functioning (Curtis, 2006). Specifically, DLPFC and FEF maintain and manipulate visuospatial information (Liu et al., 2010; Wu et al., 2014), whereas PPC stores encoded representations temporarily for future retrieval (Todd and Marois, 2004). Causal evidence of these regions’ involvements in VSWM comes from non-invasive transcranial direct current stimulation (tDCS) studies that showed positively charged anodal current over the PPC and DLPFC would produce improved visual memory of spatial locations (Tseng et al., 2012; Hsu et al., 2014) and improved control and resistance against distractors and interference (Wu et al., 2014), respectively. TDCS, in short, is a non-invasive neuromodulation technique that provides polarity-specific low-amplitude constant electrical current stimulation over targeted brain region to modulate cortical excitability and neuroplasticity (Nitsche and Paulus, 2001; Fritsch et al., 2010). It is generally agreed that anodal (positively charged) stimulation produces an excitatory effect to the targeted cortex, while the cathodal (negatively charged) stimulation gives an inhibitory effect, though there are many other factors that contribute to the final behavioral outcome (Fertonani and Miniussi, 2016).

In the present study, we aim to employ tDCS to facilitate VSWM in patients with DPN. Previously we have reported that anodal tDCS over the right DLPFC is able to improve healthy young adults’ VSWM span when complex manipulation of information is involved. Specifically, when participants have to retrieve VSWM information in a backward manner while trying to ward off motor interference and distractions, anodal tDCS is able to improve DLPFC functioning and decrease the negative impact of backward recall and motor interference altogether (Wu et al., 2014). These findings have great therapeutic implications for neurological populations who may suffer from VSWM deficits, such as patients with DPN. However, whether or not such effect from young healthy individuals can be readily generalized or transferred to neurological populations remains to be tested. It is entirely possible that, the neurological population, whose VSWM decrement tends to be caused by structural impairment or neuronal degeneration, may not be able to experience the same type of tDCS-induced improvement that was observed from neurologically intact young participants (because tDCS is thought to act on neural oscillations and functional connectivity, rather than structure changes; e.g., Yu et al., 2015). This healthy-to-neurological generalization will also have tremendous impact on the clinical feasibility of tDCS as a tool for neuro-rehabilitation. To this end, in the present study we recruited 16 patients with DPN and administrated a computerized Corsi block tapping task with varying levels of difficulty, with or without anodal tDCS in a within-subject design to investigate the potential benefits of tDCS in DPN patients while controlling for within-subject practice effects.

## Materials and Methods

### Ethical Standards

This study has received the human study approval (A-BR-101-123) from the Institutional Review Board, National Cheng Kung University Hospital. It has been carried out in accordance with The Code of Ethics of the World Medical Association (Declaration of Helsinki).

### Participants

Eighteen DPN patients (40–69 years old) were recruited for the study, and two were excluded due to inadequate visual acuity and hand tremor. Sixteen age- and education-matched healthy controls were also recruited to match the remaining 16 patients in this study. DPN patients were recruited from the Neurology Outpatient Departments at National Cheng Kung University Hospital (NCKUH) and NCKUH Dou-Liou branch. Type 2 diabetes mellitus (T2DM) was diagnosed according to the American Diabetes Association diagnostic criteria (2012). DPN was diagnosed by experienced neurologists based on “confirmed diabetic sensorimotor polyneuropathy” according to the Toronto classification of distal symmetric diabetic polyneuropathies (Tesfaye et al., 2010) in which patients show symptoms or signs of polyneuropathy and have abnormality of nerve conduction velocity (NCV). The severity of DPN enrolled in this study was grade 2a or grade 2b by Dyck’s staging rule (Dyck et al., 2011) that required the presence of abnormal NCV, and typical neuropathic symptoms (with or without signs, grade 2a) and a moderate degree of weakness of ankle dorsiflexion (with or without typical neuropathic symptoms, grade 2b). Subjects with other conditions that might affect their cognitive functions other than diabetic neuropathy, such as those with brain structural lesions, neurological disorders (stroke, seizure, parkinsonism, dementia, traumatic brain injury, brain tumor, and encephalitis), metabolic encephalopathy, or use of sedative medication or those who were not suitable for the VSWM task and tDCS application, were excluded from enrollment. All subjects gave informed consent prior to participation in the study. This study was approved by the NCKUH Institutional Review Board of Human Study.

### Corsi Block Tapping Task (CBT)

**FIGURE 1:**
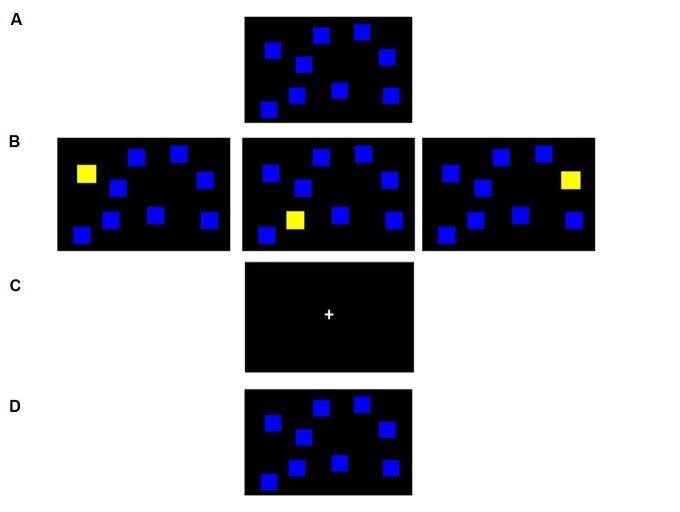
**Computerized Corsi block tapping task (CBT).** In each trial, nine blue squares are randomly placed on the screen as placeholders **(A)**. In the encoding phase **(B)**, some of the squares would change color from blue to yellow in a sequential manner and participants are asked to memorize the location and the sequence of such presentation. During the 5-s delay retention phase **(C)**, participants perform the modified Luria manual sequencing task in the interference condition, vs. no manual task in the non-interference condition. Finally, in the recall phase **(D)**, participants are to point out the squares that changed color in the correct locations either in a forward or backward manner.

The Corsi block tapping task (CBT) had been used to measure VSWM capacity since 1972. Participants were asked to memorize and recall the locations and sequence of displayed visual targets either in forward or backward manner (Fischer, 2001). In the beginning, nine placeholders, in the form of nine blue squares, were displayed in random locations on the screen as background (**Figure 1A**). During the encoding phase (**Figure 1B**), yellow target squares would appear, each for 500 ms, in randomized sequence. During the 5-s delay phase (**Figure 1C**), participants were asked to do either nothing (for non-interference condition) or the modified Luria manual sequencing task (for interference condition; Weiner et al., 2011). After a 5-s delay was the recall phase (**Figure 1D**), where participants were presented with the initial nine-blue-block background and were asked to point out the target squares in the same order as originally presented (forward condition), or in reversed sequence (backward condition). The stimuli were displayed on a 22-inch touch screen, and from which the responses were obtained. The paradigm was computer-adaptive, and thus the set size would only increase if the participants actually passed two consecutive trials of the same span. The test started from 2 targets and went up to 9 as maximum. If the participant failed two consecutive trials at one specific span, the session was terminated at that span level, which defined the individual’s VSWM span. Each CBT session consisted of four conditions that included forward recall (recalling the sequence order as it appeared), forward recall with interference (forward recall after performing a motor interference task during the retention delay phase), backward recall (recalling the stimuli sequence in reversed order), and backward recall with interference, sequentially.

### Experiment Design and Procedures

Both DPN patients and healthy control subjects received brain CT, NCV, Wechsler Adult Intelligence Scale, 4th edition (WAIS-IV), Montreal Cognitive Assessment-Taiwan version (MOCA), and serum tests (FS sugar, HbA1C, Bun/Cr and eGFR, ALT/ALT, Vitamin B12, syphilis VDRL, TSH/fT4/T3, cortisol level) to determine the DM state and to exclude other metabolic disorders that would otherwise affect cognition. The baseline cognitive assessments by WAIS-IV and MOCA of DPN patients were compared with their age- and education-matched healthy counterparts. After the baseline screen and an evaluation by an experienced neurologist to confirm the diagnosis of DPN, DPN patients participated in two different CBT sessions: active and sham tDCS, which took place on two different days at least 24 h apart, with counter-balanced order across participants to avoid tDCS after-effect and practice effect, if any. On both tDCS and sham days, the experiment began with a session of pre-treatment CBT, followed by tDCS or sham treatment, and again followed by another post-treatment CBT (**Supplementary Figure S1**). This pre–post design on the sham day would give us each participant’s normal improvement level due to practice, thereby controlling for any practice effects that may occur. In other words, patients’ pre–post improvement on the tDCS day, if any, must outperform their own improvement from the sham day in order to demonstrate a significant effect of tDCS. The sequence of the four conditions of CBT (forward, forward with interference, backward, and backward with interference) was the same for all sessions. All participants had the introduction and full practice to familiarize themselves with the four CBT conditions prior to the formal experiment. The practice CBT was then performed after the familiarization, and participants whose scores did not fluctuate (+2 or -2) in memory span in all four conditions were considered as well-practiced.

### Anodal tDCS Protocol

Right DLPFC was located as F4 according to the international 10–20 EEG system (e.g., Herwig et al., 2003). Anodal tDCS (NeuroConn Eldith DC-Stimulator) was delivered over F4 and the cathodal pole over the left cheek to avoid contaminating other brain regions during electrical stimulation (Hsu et al., 2011; Wu et al., 2014). Electrical current was applied via a pair of rubber electrodes (5 cm × 7 cm) housed in saline-soaked sponge coverings (6.5 cm × 7.5 cm). The direct current was applied with 2 mA for 15 min (2 mA/6.5 cm × 7.5 cm = 0.041 mA/cm^2^) which could facilitate VSWM in healthy adults (Tseng et al., 2012; Wu et al., 2014) and create an excitatory effect for up to 90 min (Nitsche and Paulus, 2001). The sham condition followed identical tDCS procedures except that the stimulation only lasted 30 s.

## Results

### Baseline Characteristics

**FIGURE 2:**
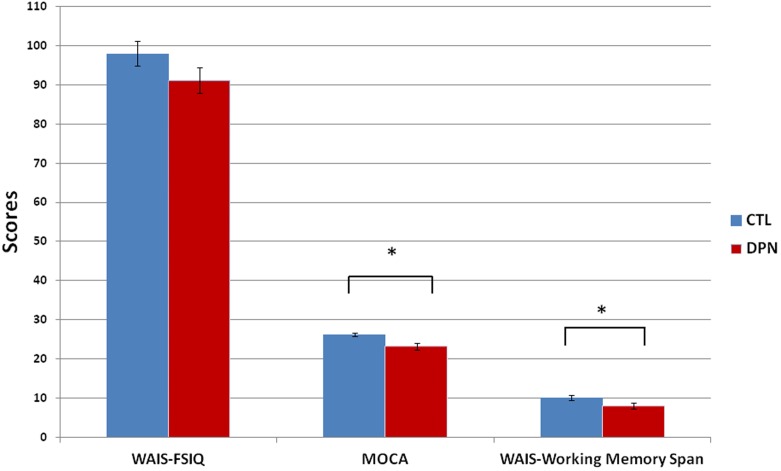
**Baseline intelligence.** The DPN patients had no significant difference in general intelligence compared with the control subjects (WAIS-FSIQ, mean ± SE: DPN 91.2 ± 3.3 vs. CTL 98.0 ± 3.2, *p* = 0.096) but the patients did show lower scores in MOCA (mean ± SE: DPN 23.2 ± 0.9 vs. CTL 26.2 ± 0.5, *p* = 0.029) and forward-backward digit span in WIAS (mean ± SE: DPN 8 ± 0.7 vs. CTL 10.1 ± 0.6, *p* = 0.034). ^∗^*p* < 0.05. SE, standard error; WAIS, Wechsler Adult Intelligence Scale; FSIQ, full scale intelligence quotient; MOCA, Montreal Cognitive Assessment; DPN, patients with diabetic polyneuropathy; CTL, age- and education-matched healthy control.

Paired-sample *t*-test were conducted to analyze the characteristics and the differences between DPN patients (*n* = 16, 14 males and 2 females) and their healthy age- and education- matched counterparts (*n* = 16, seven males and nine females; **Table 1**). Patients with DPN showed significantly higher AC sugar (DPN 184 ± 22.4 vs. control 94 ± 2.3, *p* = 0.001, *d* = 1.459, normal range 70–100 mg/dl) and HbA1C (DPN 8.6 ± 0.4 vs. control 5.5 ± 0.1, *p* < 0.001, *d* = 2.745, normal range 4–6%) over healthy controls. Control subjects showed normal NCV (average of all the sensory and motor NCVs from four limbs) while DPN patients had significant lower NCV than controls (DPN 41 ± 1.8 vs. control 53 ± 0.7 m/s, *p* < 0.001, *d* = -2.269) and below the normal reference value. General intelligence was measured by the full scale intelligence quotient (FSIQ) in WAIS-IV (Wechsler, 2008), and we found no difference between patients and controls (DPN 91.2 ± 3.3 vs. control 98 ± 3.2, *p* = 0.096) except for lower forward-and-backward digit span in the patients (DPN 8 ± 0.7 vs. control 10.1 ± 0.6, *p* = 0.034, *d* = -0.832). Frontal lobe-dependent cognitive functions were evaluated by MOCA (Nasreddine et al., 2005), and the patients showed significantly lower score than controls (DPN 23.2 ± 0.9 vs. control 26.2 ± 0.5, *p* = 0.029, *d* = -1.064) and below the cutoff score 26 for mild cognitive impairment (MCI; **Figure 2**). Among the DPN patients, there was no significant gender difference on the pre-sham CBT (Male 4.4 ± 0.15 vs. Female 3.6 ± 0.38, *p* = 0.084), pre-tDCS CBT (Male 4.4 ± 0.17 vs. Female 3.9 ± 0.35, *p* = 0.314), post-sham CBT (Male 4.4 ± 0.15 vs. Female 3.9 ± 0.35, *p* = 0.243) and post-tDCS CBT (Male 4.8 ± 0.15 vs. Female 4.1 ± 0.22, *p* = 0.112) by paired-sample *t*-test.

**Table 1:** Baseline characteristics of subjects.

	DPN	CTL	*p*-value
Age (year)	57.6 ± 2.3	56.8 ± 2.1	0.786
Education (year)	9.4 ± 0.7	9.4 ± 0.6	1
NCV (m/s)	41 ± 1.8	53 ± 0.7	<0.001^†^
AC sugar (mg/dl)	184 ± 22.4	94 ± 2.3	0.001^†^
HbA1C (%)	8.6 ± 0.4	5.5 ± 0.1	<0.001^†^
FSIQ WAIS	91.2 ± 3.3	98 ± 3.2	0.096
Working memory span in WAIS	8 ± 0.7	10.1 ± 0.6	0.034^†^
MOCA	23.2 ± 0.9	26.2 ± 0.5	0.029^†^


*Values are mean ± standard error, and *t*-test *p*-value.*

**p*-value^†^ represents *p*-value < 0.05.*

*NCV, nerve conduction velocity; AC sugar, Ante Cibum sugar; HbA1C, hemoglobin A1C; FSIQ, full scale intelligence quotient; WAIS, Wechsler Adult Intelligence Scale; MOCA, Montreal Cognitive Assessment; DPN, patients with diabetic polyneuropathy; CTL, control subjects.*

### CBT Performances

The DPN patients’ CBT spans (forward recall, forward with interference, backward recall, backward with interference) before and after sham and verum tDCS were analyzed with a four-way repeated-measures ANOVA, with the factors of directionality (forward vs. backward), interference (with vs. without interference), day (tDCS day vs. sham day), and pre–post (pre- vs. post-treatment). There was a significant main effect of interference [*F*(1,15) = 67.262, *p* < 0.001, η^2^ = 0.818], day [*F*(1,15) = 8.378, *p* = 0.011, η^2^ = 0.358], and pre–post [*F*(1,15) = 9.060, *p* = 0.009, η^2^ = 0.377], but no significant main effect of directionality [*F*(1,15) = 0.697, *p* = 0.417]. Comparing the means revealed that the patients’ CBT performance was (1) better without concurrently motor interference (span 4.977 ± 0.245) than with motor interference (span 3.813 ± 0.191), (2) better on the day of tDCS (span 4.492 ± 0.211) than the day of sham (span 4.297 ± 0.210), and (3) better in the post-treatment session (span 4.500 ± 0.200) than pre-treatment (span 4.289 ± 0.221).

**FIGURE 3:**
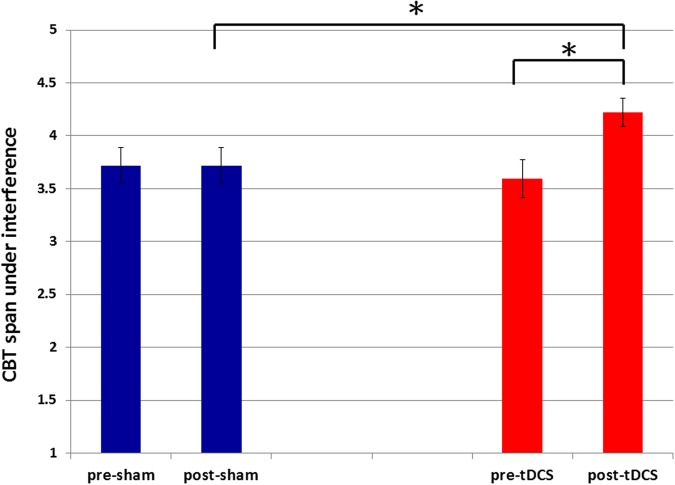
**Pre–post sham and tDCS comparisons of CBT span under interference.** The CBT spans in the motor interference condition were significantly improved (*p* = 0.007) after tDCS treatment (span mean ± SE: 4.219 ± 0.133) relative to the pre-tDCS session (span mean ± SE: 3.594 ± 0.179) on the same day. This effect was not observed between pre- (span mean ± SE: 3.719 ± 0.169) and post-sham (span mean ± SE: 3.719 ± 0.169) performance (*p* = 1.000) on the sham day. The CBT spans in the interference condition were significantly increased (*p* = 0.023) when comparing the post-tDCS block (span mean ± SE: 4.219 ± 0.133) with the post-sham block (span mean ± SE: 3.719 ± 0.169) spans. There was no significant difference (*p* = 0.614) between the pre-tDCS block (span mean ± SE: 3.594 ± 0.179) and the pre-sham block (span mean ± SE: 3.719 ± 0.169). SE represents standard error. ^∗^*p* < 0.05.

Besides main effects, we also observed a significant three-way interaction between day, pre–post and interference [*F*(1,15) = 10.058, *p* = 0.006, η^2^ = 0.401] and a significant two-way interactions between day and pre–post [*F*(1,15) = 7.757, *p* = 0.014, η^2^ = 0.341]. No significant four-way interaction was observed between day, pre–post, interference and directionality [*F*(1,15) = 0.348, *p* = 0.564]. Separate comparisons showed that the two-way interaction between day and pre–post was mainly driven by a bigger pre–post difference on the tDCS day (span, pre-tDCS 4.297 ± 0.157 to post-tDCS 4.688 ± 0.133, *p* = 0.06, *d* = 0.694) over the sham day (span, pre-sham 4.281 ± 0.143 to post-sham 4.313 ± 0.141, *p* = 0.877). Furthermore, such pre–post improvement on the tDCS day was most notable when dealing with motor interference (span, pre-tDCS 3.594 ± 0.179 to post-tDCS 4.219 ± 0.133, *p* = 0.007, *d* = 1.023) rather than without interference (span, pre-tDCS 5.000 ± 0.191 to post-tDCS 5.156 ± 0.201, *p* = 0.575). The same comparisons did not show statistical significance in the sham-day condition (CBT span with motor interference, pre-sham 3.719 ± 0.169 vs. post-sham 3.719 ± 0.169, *p* = 1.000; **Figure 3**). Thus, there was not even a practice effect in DPN patients when sham tDCS was applied. This discrepancy over the presence and absence of an improvement effect (that was unique to post-tDCS CBT with motor interference) was the driving factor behind the three-way interaction between day, pre–post and interference. Similar comparisons can also be done between post-treatment interference spans across days (tDCS vs. sham), which would yield similar results (CBT span with interference, post-sham 3.719 ± 0.169 vs. post-tDCS 4.219 ± 0.133, *p* = 0.023, *d* = 0.849) as the *post hoc* comparisons we’ve done above. Also note that there was no difference between the baseline pre-treatment sessions (CBT span with interference, pre-sham 3.719 ± 0.169 vs. pre-tDCS 3.594 ± 0.179, *p* = 0.614; **Figure 3**). Together, we observed an improvement effect in patients’ CBT performance when motor interference was involved, and only when tDCS was applied.

### Correlation of VSWM and NCV

**FIGURE 4:**
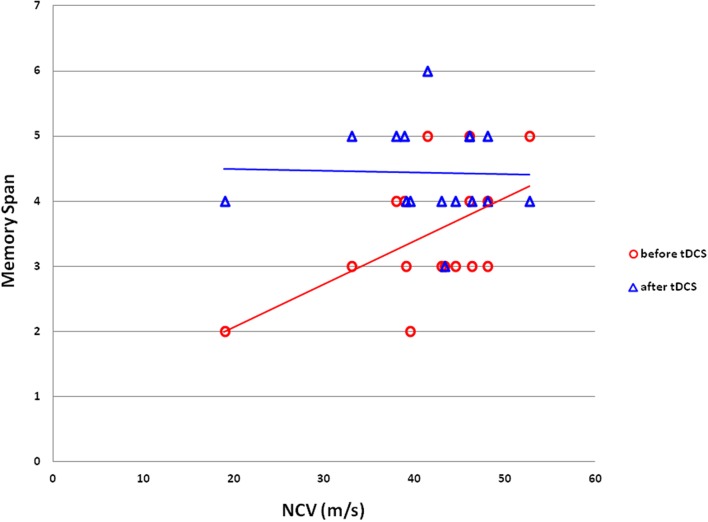
**Correlation between VSWM span and NCV in DPN patients.** VSWM span from the backward-recall-with-interference CBT was significantly correlated with peripheral neuropathy state measured via NCV in the DPN patients (Pearson correlation: 0.530, *p* = 0.035; red line). The correlation diminished (blue line) after DLPFC tDCS (Pearson correlation: -0.026, *p* = 0.922) due to the improvement of VSWM span in the lower-performing patients. VSWM, visuospatial working memory; NCV, nerve conduction velocity; DPN, diabetic polyneuropathy; DLPFC, dorsolateral prefrontal cortex; tDCS, transcranial direct current stimulation.

To explore the possibility of a relationship between patients’ peripheral neuropathy state and their VSWM capacity, we plotted each patient’s CBT performance against their NCV measure (**Figure 4**). There was a positive correlation between patients’ NCV and their baseline backward-with-interference CBT performance on the tDCS day (Pearson correlation: 0.53, *p* = 0.035, *r*^2^ = 0.281). Patients with lower NCV, which indicated worse peripheral neuropathy state, had a lower baseline capacity in VSWM before tDCS treatment when performing backward-recall CBT with Luria’s motor interference — the most difficult CBT condition of the present experiment. Importantly, this correlation diminished after tDCS treatment (Pearson correlation: -0.026, *p* = 0.922), which was mainly driven by the elevated performance from the low performers (**Figure 4**).

## Discussion

### Baseline Characteristics

The DPN patients’ general intelligence scores on WAIS were not significantly different from the age- and education-matched healthy subjects (**Figure 2**). This observation is consistent with previous reports that the estimated IQ of T2DM patients is on par to healthy controls both at baseline and after a 4-year follow-up (van den Berg et al., 2010). However, in terms of digit span, a sub-item of WAIS-IV that measured verbal WM capacity demanding participants to recall series of numbers in a forward and backward manner, DPN patients’ performance was significantly lower than the healthy controls (**Figure 2**). This implies that perhaps cognitive decline is already taking place in these DPN patients, but at a finer level that is specific to attention and WM, and thus cannot be reflected via a domain-general IQ test. This is somewhat supported by our findings in MOCA, a cognitive screening tool assessing multiple aspects of cognitive functioning, especially frontal lobe-dependent functions such as executive functions and WM. We observed lower MOCA scores in DPN patients (DPN 23.2 vs. CTL 26.2, *p* = 0.029) that was below the cutoff score 26 for MCI (**Figure 2**). Gender difference on the change of motor cortical excitability has been reported in young athletes after exercise (Perciavalle et al., 2010). However, there was no significant sex difference on the pre- or post-treatment CBT of DPN patients in this study. Together, these baseline characteristics suggest that although general intelligence was not significantly affected in DPN patients yet, our DPN patients did have emerging signs of MCI with WM deficit.

### Link between Peripheral and Central Neuropathy

One of the novel observations from the present study is the positive correlation between patients’ NCV and baseline backward interference CBT performance (**Figure 4**). This positive relationship between VSWM capacity (i.e., backward interference CBT spans) and the severity of peripheral neuropathy (i.e., NCV values) provides a link between diabetic manifestations of peripheral and central neuropathies. There has been some proposed causes for cognitive impairments in diabetic patients, including microvascular disease, advanced protein glycation, mitochondrial dysfunction and oxidative stress, insulin resistance and the abnormal deposition of Aβ and tau phosphorylation, brain blood barrier dysfunction, impaired neruogenesis and inflammation problems (Exalto et al., 2012; Umegaki, 2014). Several pathways have also been identified as responsible for diabetic peripheral neuropathy; for example, excess glycolysis, overload of the mitochondrial electron transport chain, increased oxidative stress, inflammatory injury, insulin resistance, and impaired insulin signaling (Vincent et al., 2011; Kim and Feldman, 2012). However, whether diabetic peripheral neuropathy shares some overlapping mechanisms with central neuropathy remains unknown, though our findings do suggest a co-existence and correlation at the very least.

Here, it is important to note the discrepancy between the severity of the patients’ central and peripheral neuropathy. We selectively enrolled DPN patients with severe peripheral neuropathy at Dyck’s grade 2a or 2b, who suffered from typical neuropathic symptoms and even with distal limb weakness (Tesfaye et al., 2010). However, these patients showed only mild cognitive deficit on MOCA (23.2 out of total score 30, cutoff score 26 for MCI) and digit span, without obvious decline in general intelligence (**Table 1**). In the condition of diabetic pathophysiology, it is hypothetically reasonable that the peripheral neuropathic changes would manifest more prominently than the central neuropathic changes because a distal and isolated nerve fiber is more vulnerable than a network of neural circuits. However, at the higher cognitive level, VSWM seems to decline along with the evolution of peripheral neuropathy, and reflect the cognitive change associated with DPN progression in a positive correlation manner (**Figure 4**). Therefore, in addition to the co-existence and correlation between central and peripheral neuropathy, our data also suggest a discrepancy in severity of neuropathic symptoms, among which the peripheral one progresses faster than the central one.

Recent studies have shown that patients with T2DM have a higher risk of developing cognitive impairment and are prone to dementia (Ott et al., 1999; Stewart and Liolitsa, 1999; Exalto et al., 2012; Pearce et al., 2012). A 5.5-year follow-up study showed that DM patients have a 65% increase in the risk of developing Alzheimer’s disease (AD) compared with those without DM (Arvanitakis et al., 2004). The Honolulu-Asia Aging Study also showed a 1.8-fold higher risk for AD and a 2.3-fold higher risk for vascular dementia (VD) in T2DM patients (Peila et al., 2002), and the Hisayama study showed that diabetic patients have 1.74-fold higher risk of all-cause dementia and 2.05-fold higher risk of AD compared to those without DM (Ohara et al., 2011). The pooled relative risk of AD is 1.46 and the relative risk of VD is 2.5 in subjects with T2DM based on a comprehensive meta-analysis of population-based longitudinal studies (Cheng et al., 2012). The clinical relevance of the cognitive deficit and WM impairment shown in our study lies in its role as an early warning sign of central neuropathy in DPN patients. Indeed, VSWM has been shown to be specifically effective in the early detection of dementia and AD. One notable study (Parra et al., 2010) found that participants’ VWSM performance can serve as a preclinical behavioral marker to separate asymptomatic carriers of familial AD and healthy control subjects, while other standardized neuropsychological tests (e.g., Mini-Mental State Examination, verbal fluency, Rey–Osterrieth complex figure copy test, Wisconsin card sorting, etc.) cannot. It is quite common for patients with advanced DPN to seek medical help for the sensorimotor symptoms of DPN but fail to notice the emerging patterns of MCI. Our data therefore suggest that VWSM may be a useful tool for early detection of central neuropathy and cognitive decline in DPN patients, and its decline could be an early red flag sign to those DPN patients who will potentially progress from MCI to dementia in the chronological spectrum of the disease.

### The Effect of DLPFC tDCS on VSWM

In this study we observed a facilitating effect of anodal tDCS over right DLPFC in memory span (**Figure 3**). This effect was only observed in the interference conditions, which is consistent with our previous findings (Wu et al., 2014). In our previous study, we found specific tDCS effect in young adults only in the backward-with-interference condition, the most difficult condition of all four. We concluded that only the task condition with heaviest DLPFC involvement (e.g., warding off motor interference and recall in a reverse sequence), that was also difficult enough for our young adults, would show a tDCS-induced facilitation because it is the actual condition that people have room for improvement. Therefore, the more difficult the task is (i.e., backward recall with interference), the more ability is required, for which the participants could really use the facilitation brought forth by anodal tDCS. Compared with our patients in the present study, it is evident that regardless of forward and backward recall, the addition of motor interference was already difficult enough for the patients. Therefore, it is reasonable that the effect of tDCS would be more generalized to be observed in all conditions with motor interference. The cognitive stage that was bolstered by tDCS is likely the retention period, since that is when motor interference took place. This is also consistent with previous study that suggested the age range from 40 to 69 years is the most vulnerable to interference effects during the maintenance phase of WM task (McNab et al., 2015).

Regarding the beneficial effect of tDCS, it is important to note the interaction between tDCS and natural individual differences in VSWM. From **Figure 4** it is evident that the disappearance of the positive correlation between NCV and VSWM was mainly driven by an improvement in those low-performers whose original VSWM span fell below 4. The high-performers also showed improvement, which was less in magnitude when compared with the low-performers. This interaction between tDCS and one’s baseline performance is consistent with previous studies, where young healthy participants whose VSWM fell below the median split tend to benefit the most from current stimulation (Tseng et al., 2012; Hsu et al., 2014; Heinen et al., 2016). For instance, Tseng et al. (2012) and Hsu et al. (2014) reported that tDCS over the right parietal cortex can improve VSWM recognition performance, but only in participants whose memory span was lower than average to begin with. Heinen et al. (2016) also found similar effect in low-performers on VSWM precision. Thus, it appears that the high-performers have reached their maximum potential at baseline, and adding tDCS to the mix is not enough to break such cognitive ceiling. Although, some may argue that if the high-performers are properly challenged to the maximum of their cognitive capacity, perhaps the facilitative effect of tDCS will emerge as observed in the low-performers. It should be noted that in the current task, none of the patients had hit the CBT ceiling of nine blocks, with the best score of all being 6. Therefore, the CBT was definitely challenging enough, but 15 min of stimulation simply cannot give the high-performers what they do not already have.

To contextualize our findings within the general framework of attention and VSWM, we think that the right DLPFC is mainly involved in top–down control for VSWM (e.g., Yang et al., 2012), which belongs to the central executive for managing concurrent interference rather than the bottom–up slave systems (i.e., memory storage). This would explain why PPC is usually implicated in the VSWM literature in studies that manipulate memory load but not levels of general interference. Therefore, the forward-backward and interference manipulations employed here are both taxing on the frontal cortex, giving tDCS a chance to facilitate in the tougher conditions. These results also indicate that the cross-domain motor interference may impede VSWM performance at the central executive level that involves the right DLPFC, thus the facilitative effect of tDCS can be used to overcome the gap between patients’ ability and task requirement.

### tDCS Focality and Network Functional Connectivity

The effect of tDCS reported here in protecting memory retention against cross-modal interference and shifts of attention is consistent with our current understanding of DLPFC functioning, such as ignoring distraction, information updating, monitoring, attentional regulation and manipulation, or dual-tasking (Toepper et al., 2010). Therefore, it is reasonable to assume that a big part of the tDCS effect here is a result of tDCS-induced elevation in DLPFC activity. This is not to claim, however, that the reported effects here are solely due to DLPFC activation alone. Indeed, the focality and network effect of tDCS have been under rigorous investigation in recent years. In terms of focality, in most tDCS studies, the size of the tDCS sponge coverings (6.5 cm × 7.5 cm) can be larger than the exact size of the region of interest. Also, not all current passes through the scalp, and even when it does, current inside the skull can be diffused by CSF. Therefore, in the present study we do not intend to rule out the possibility that perhaps areas adjacent to DLPFC may also have received stimulation of tDCS.

Recent studies that combined tDCS and neuroimaging techniques have also reported an increased functional connectivity between the tDCS-stimulated region and other regions that may be involved in the task at hand, generating a network-wide activation (Keeser et al., 2011). We used 2 mA to modulate the prefrontal cortex function based on one particular fMRI-tDCS study that showed 2 mA anodal tDCS not only increased the BOLD responses of the stimulated target and nearby regions, it also promoted the functional connectivity between these areas with other areas that are relevant to the behavior task (Yu et al., 2015). Therefore, in the context of the present experiment it is highly possible that our patients may have recruited more areas beyond DLPFC within the VSWM network such as PFC, PPC, and the premotor cortex (Toepper et al., 2010) after tDCS application and resulted in VSWM improvement. Although, we have observed a facilitatory effect of tDCS over the DLPFC in improving VSWM, it is important to note that the precise directionality and focality of tDCS should be cautiously taken into consideration when interpreting the results. In terms of directionality, although we have previously observed that 20 min of 2 mA stimulation can improve cognitive performance and increase BOLD signals in the stimulated region (Yu et al., 2015), it has also been reported that the bidirectional effect of anodal and cathodal stimulation can reverse directions when stimulated for 20 min in the motor cortex (Batsikadze et al., 2013). Therefore, it remains possible that the observed effect in the present study may be a result of increased inhibition instead of increased facilitation. In addition, in terms of tDCS focality, although highest current density does occur beneath the target and reference electrodes, regions that are phase coherent (Tseng et al., 2016), adjacent (Sadleir et al., 2010), functionally connected (Yu et al., 2015), signally complex (Liang et al., 2014), or in between the electrodes (Datta et al., 2009) can also show higher current densities or activation. Therefore, the focality or the current spread of tDCS can be interactive with the location and distance between the electrodes (Bikson et al., 2010; Faria et al., 2011). As such, the issues of directionality and focality of tDCS is still limitation of the present study and requires further research. In addition, in the present study we have demonstrated an effect of tDCS via behavioral VSWM performance, but the underlying neural mechanisms, particularly for the populations with neurological deficits and how tDCS interacts with a pathological brain, still remains a critical question and warrants more neurophysiological (e.g., electroencephalogram and evoked potential) and functional imaging studies.

## Conclusion

This study showed that DPN patients with severe peripheral neuropathy presented merely MCI and WM decline, implying the co-existence of peripheral and central neuropathic changes in DM patients with discrepancy of severity. Even when DPN patients may have the same general intelligence as their healthy counterparts, VSWM can be used as an early behavioral marker of imminent cognitive decline. We found a positive correlation between patients’ NCV level and VSWM span in the backward-with-interference condition, suggesting a link between peripheral and central neuropathies. Most importantly, anodal tDCS over the right DLPFC improved low-performers’ memory span to be on par with the high-performers. Together, these findings serve as a reminder for clinicians of the subclinical cognitive declines in patients with DPN, and strike new insight of the therapeutic potential of tDCS for DPN-related VSWM deficits.

## Author Contributions

Y-JW has designed and executed the study, collected data, and wrote the manuscript. Y-JW and PT performed the statistical analysis. PT, C-CL, K-SH, and C-HJ provided suggestions on the study design. PT and C-CL advised the data analysis and revised the manuscript. Y-JW, H-WH, and C-CL were in charge of the patient enrollment and clinical examination. J-FH supervised the neuropsychological tests.

## Conflict of Interest Statement

The authors declare that the research was conducted in the absence of any commercial or financial relationships that could be construed as a potential conflict of interest.

## Supplementary Material

**FIGURE S1 | Schematic diagram of the experimental sessions.** DPN patients participated in two experiments, tDCS vs. sham, on two different days in a counter-balanced order and at least 24 h apart. The experiments began with a session of pre-treatment CBT, followed by tDCS or sham treatment, and again followed by another post-treatment CBT to control for any practice effect beyond the effect of brain stimulation.
