# Supplementary figures and images for: The Facilitative Effect of Transcranial Direct Current Stimulation on Visuospatial Working Memory in Patients with Diabetic Polyneuropathy: A Pre–post Sham-Controlled Study
Source: Front Hum Neurosci. 2016 Sep 28;10:479. doi: 10.3389/fnhum.2016.00479 (PMC5039168; doi:10.3389/fnhum.2016.00479)

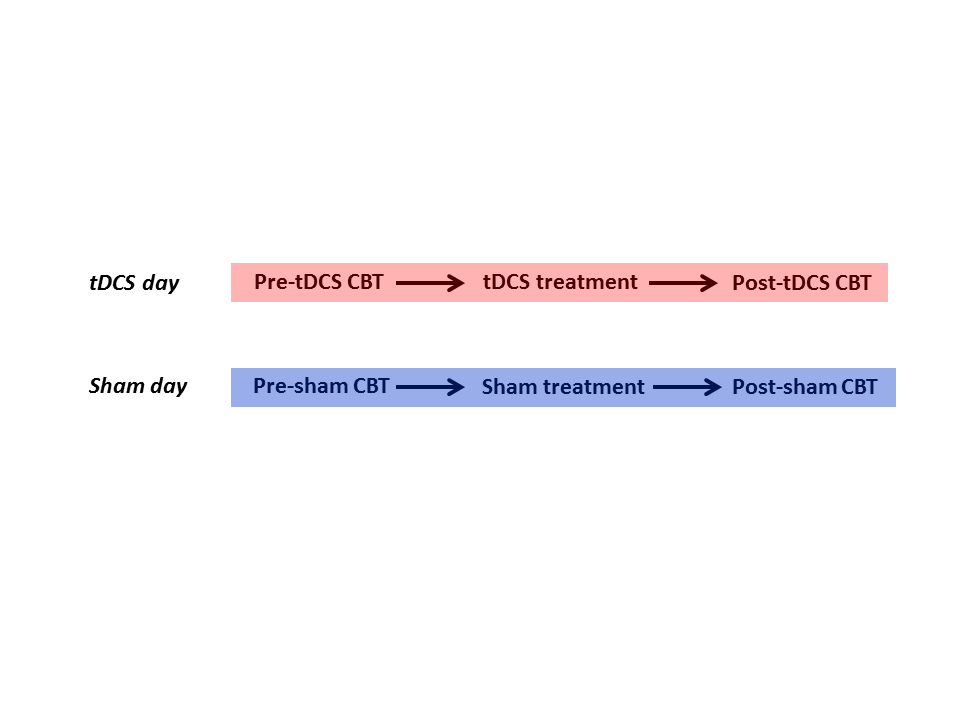

Supplement: Supplementary file 1 [file Image_1.TIF]
